# Anti-cancer effects of disulfiram in head and neck squamous cell carcinoma via autophagic cell death

**DOI:** 10.1371/journal.pone.0203069

**Published:** 2018-09-13

**Authors:** Young Min Park, Yoon Young Go, Sun Hwa Shin, Jae-Gu Cho, Jeong-Soo Woo, Jae-Jun Song

**Affiliations:** 1 Department of Otorhinolaryngology, Yonsei University College of Medicine, Seoul, Korea; 2 Department of Otorhinolaryngology-Head and Neck Surgery, Korea University College of Medicine, Seoul, Korea; University of South Alabama Mitchell Cancer Institute, UNITED STATES

## Abstract

**Background:**

Disulfiram (DSF), which is used to treat alcohol dependence, has been reported to have anti-cancer effects in various malignant tumors. In this study, we investigated the anti-cancer effects and mechanism of DSF in HNSCC.

**Methods:**

Head and neck squamous carcinoma cell lines (FaDu and Hep2) were used to analyze the anti-cancer effects of DSF. The anti-cancer effects of DSF were confirmed in vivo using a xenograft tumor model.

**Results:**

The anti-cancer effects of DSF in HNSCC were found to be copper (Cu) dependent. Specifically, DSF/Cu markedly inhibited HNSCC at a concentration of 1 μM. After DSF/Cu administration, production of reactive oxygen species (ROS) was remarkable starting at 0.5 μM, suggesting that the inhibitory effects of DSF/Cu on HNSCC are mediated through the formation of ROS. The levels of phospho-JNK, phospho-cJun and phospho-p38 were increased after DSF/Cu treatment while levels of phospho-Akt were decreased. These results suggested that the inhibitory effects of DSF/Cu on HNSCC cells involve ROS formation and down-regulation of Akt-signaling. Through these molecular mechanisms, DSF ultimately induce the inhibitory effects on HNSCC cell lines mainly through autophagic cell death, not apoptotic cell death. Lastly, we investigated the clinical relevance of DSF/Cu using a HNSCC xenograft animal model, which showed that tumor growth was remarkably decreased by DSF (50 mg/kg injection).

**Conclusion:**

In treating patients with HNSCC, DSF may contribute to improved HNSCC patient’s survival. The characteristic anti-cancer effects of DSF on HNSCC may suggest new therapeutic potential for this medication in HNSCC patients.

## Introduction

There has been no significant change in the survival rate of patients with head and neck squamous cell carcinoma (HNSCC) despite the development of various treatment modalities over the past several decades. Because the overall survival rate of HNSCC patients is less than 50%, there is a significant need for development of innovative therapeutic approaches. [1] However, due to the high cost and long-time needed to develop new therapeutic drugs, the rate of new drug approvals is very low. Therefore, drug-repurposing, in which previously unknown anti-cancer effects of existing drugs are identified in other diseases can be a good alternative to development of new therapeutic drugs. Furthermore, drug-repurposing can be a faster and less costly approach to the development of new drugs because the clinical safety of existing drugs has already been demonstrated and clinically available formulations already exist. [2]

Disulfiram (DSF), which is used to treat alcohol dependence, has been reported to have anti-cancer effects in various malignant tumors in several non-clinical trials, and clinical trials for breast cancer patients have been conducted. [3–8] DSF inhibits aldehyde dehydrogenase in the liver and has also been shown to inhibit stem-like tumor-initiating cells. [9,10] Although the precise mechanism behind the observed anti-cancer effects of DSF have not yet been clarified, it has been shown that the anti-cancer effects are mediated, at least in part, by inhibition of proteasome activity and enhancing copper binding. [11–13]

Alcohol and tobacco are the main causes of HNSCC, and appropriate counseling on smoking and drinking before and after treatment are important for determining the prognosis of HNSCC patients. Thus, DSF is clinically significant in that it can be used to stop alcohol use in HNSCC patients, and may also have anti-cancer effects of it that contribute to increased patient survival. However, the anti-cancer effects and mechanism of DSF in HNSCC have not yet been studied. Thus, we investigated the anti-cancer effects and mechanism of DSF against HNSCC in order to evaluate its potential as a new drug to treat HNSCC.

## Materials and methods

### Reagents and antibodies

DSF and copper chloride (Cu) were purchased from Sigma-Aldrich (St Louis, MO, USA). Phosphatase inhibitors and protease inhibitor cocktail tablets were purchased from Roche Applied Sciences (Penzberg, GER). The following primary antibodies were used: p-JNK, JNK, p-cJun, cJun, p-p38, phospho-Akt, and total-Akt (Cell Signaling, Beverly, CA, USA). Horseradish peroxidase (HRP)-conjugated anti-rabbit IgG and anti-mouse IgG secondary antibodies were purchase from Bio-Rad Laboratories (Hercules, CA, USA).

### Head and neck cancer cell culture

Human HNSCC cell lines (FaDu and Hep2) were purchased from the American Type Culture Collection (Manassas, Va, USA). Cells were cultured in Dulbecco's modified Eagle's medium (DMEM) solution containing 10% fetal bovine serum (FBS) and streptomycin-penicillin at a concentration of 100 U/mL in a humidified atmosphere containing 5% CO_2_ at 37°C.

### Cell viability assay

Cell viability was measured using a Cell Counting Kit (CCK)-8 (Dojindo Molecular Technologies, Japan). HNSCC cell lines were seeded in 96-well plates at a density of 5×10^3^ cells/well in 1 mL medium and treated with DSF/Cu at various concentrations (0.5–5 μM). Next, 40 μL of a CCK-8 solution added and incubated for 4h. The optical density of each culture well was then measured with a microplate reader (Bio-Tek, Winooski, VT, USA) at 450 nm.

### Annexin V/PI assay

Cells were stained using a FITC-conjugated Annexin V apoptosis detection kit according to the manufacturer’s protocol. Stained cell were were analyzed by flow cytometry using a Beckman Coulter Expo.

### Western blot analysis

HNSCC cells were treated with DSF/Cu at various concentrations (0.5–2 μM). Protein extracts were prepared from HNSCC cells using radioimmunoprecipitation assay buffer. Protein concentrations were measured using the BCA Protein Assay kit provided by the manufacturer's instructors (Thermo Scientific Pierce). 10% SDS-PAGE gels were loaded with equal amounts of protein (30 μg), transferred to membranes, and incubated with primary antibodies specific for phospho-JNK (1:1000), total-JNK (1:1000), p-p38 (1:1000), phospho-JNK (1:1000), total-cJun (1:1000), or phospho-Akt (1:1000), followed by HRP-conjugated rabbit or mouse secondary antibodies (1:1000). Blots were visualized with a chemiluminescence kit (Thermo Fisher Scientific, Fremont, CA, USA) on X-ray film and measured using AlphaEaseFC software (Alpha Innotech, San Leandro, CA, USA).

### Xenograft experiments

This study was carried out in strict accordance with the recommendations in the Guide for the Care and Use of Laboratory Animals of the National Institutes of Health. The protocol was approved by the Korea University Institutional Animal Care and Use Committee (IACUC). (Protocol Number: 2017-01-3214). Endpoints were chosen to minimize potential pain for the animal. The weight of the tumor should not exceed 5% of body weight, and subcutaneous tumors were allowed until the maximum diameter was about 20 mm in 25 g mice. For the euthanasia of the mice used in the experiment, CO_2_ method was used under the approval of IACUC. The end point was determined using a scoring system for the humane endpoint criterion. Weight change, hair condition, eye and nose, and clinical symptoms were evaluated as 0–3 points. In cases with more than 8 points of this scoring system, euthanasia cases were considered. During the course of the experiment, the experimenter checked the health status of the experimental animals daily. In this study, we did not prescribe analgesics or antibiotics to relieve pain in animals. Five-week-old female BALB/c nude mice were purchased from Shizuoka Laboratory Animal Center (Shizuoka, Japan) and bred under specific pathogen-free conditions. Animals had free access to food and water and were allowed to acclimate 1 week before the start of experiments. For in vivo experiments, we assigned 5 animals per group. FaDu cells (2 x 10^6^) in 100 μl culture medium were mixed with 100 μl of Matrigel and injected subcutaneously into the right flank of 6-week-old BALB/c nude female mice. Solvent (DMSO/PBS) or DSF (50 mg/kg/day) was injected intraperitoneally 5 days per week for 2 weeks. Changes in tumor volume were continuously measured in the control and experimental groups for 2 weeks after the start of drug injections. Tumors were measured using calipers and volumes were calculated using the formula V = (Length x Width^2^)/2.

### Statistical analysis

Data were expressed as the mean ± standard deviation, and Kruskall-Wallis test and Mann-Whitney U test were used as appropriate. P-values <0.05 were considered to indicate statistical significance. Statistical analyses were performed using SPSS 20 for Windows (SPSS, Chicago, IL, USA).

## Results

### DSF induces cell death in a Cu-dependent manner in HNSCC cell lines

**Fig 1:**
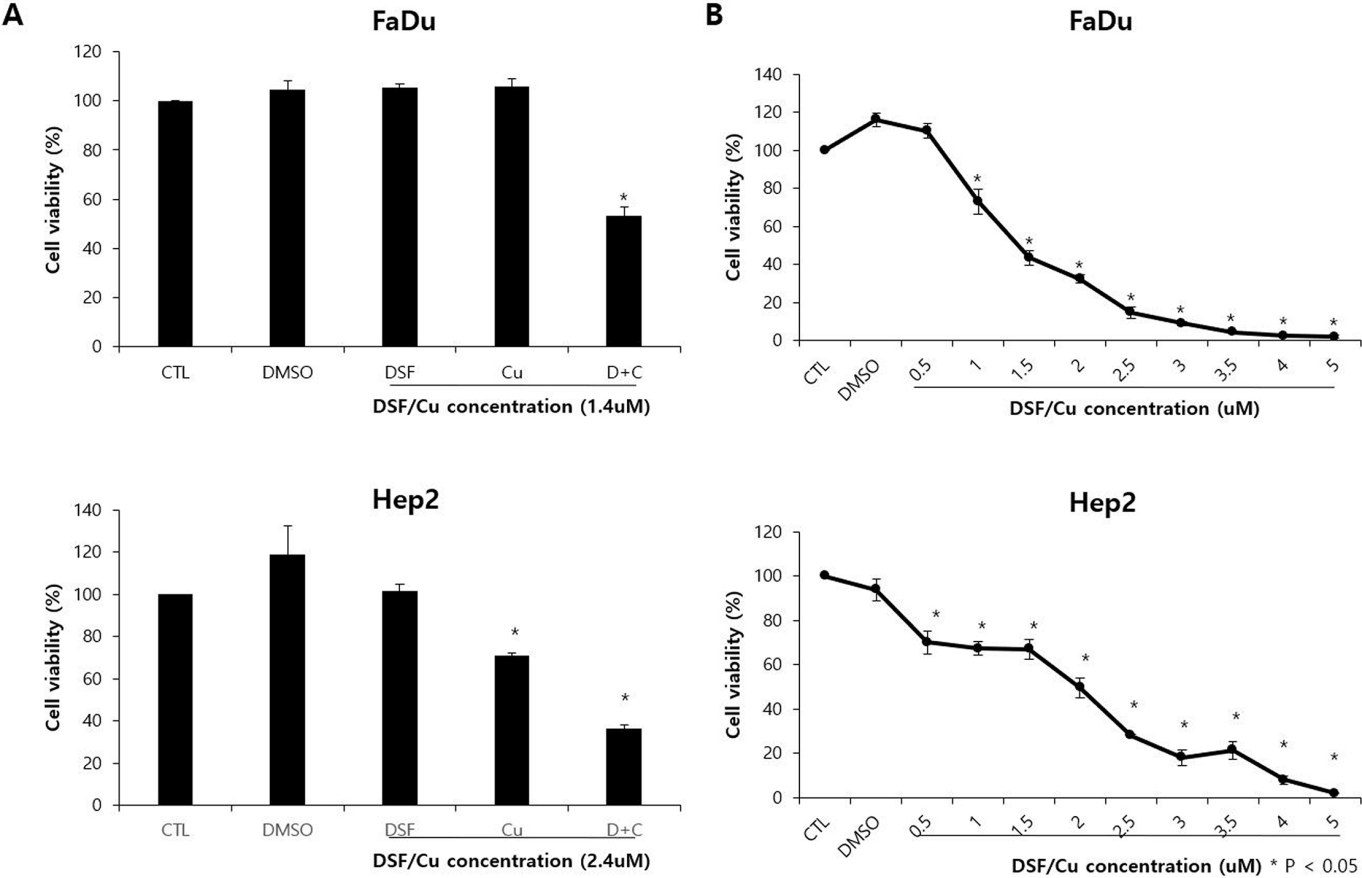
HNSCC cell lines (FaDu and Hep2) were treated with DSF at the indicated concentrations (0.5–5 μM) and cell viability was measured by CCK-8 assay. A) Degradation of cell viability by DSF was found to be dependent on copper. B) A marked decrease in viability mediated by DSF was noted at 1 μg/mL concentration in FaDu cells and at 0.5 μg/mL in Hep2 cells. Data are expressed as the mean ± standard deviation of three independent experiments. *p<0.05.

According to CCK-8 assays, DSF induced cell death in HNSCC cell lines in a Cu-dependent manner. (Fig 1A) Specifically, DSF alone did not induce cell death in HNSCC cell lines, whereas DSF combined with Cu significantly induced cell death. DSF/Cu significantly reduced the viability of HNSCC cells in a dose-dependent manner. (Fig 1B) In the FaDu cell line, a statistically significant cytotoxic effect of DSF was observed starting at a concentration of 1 μM. (p<0.05) In the Hep2 cell line, a statistically significant cytotoxic effect of DSF was observed at a concentration of 0.5 μM. (p<0.05)

### DSF induces cell death by producing reactive oxygen species (ROS)

**Fig 2:**
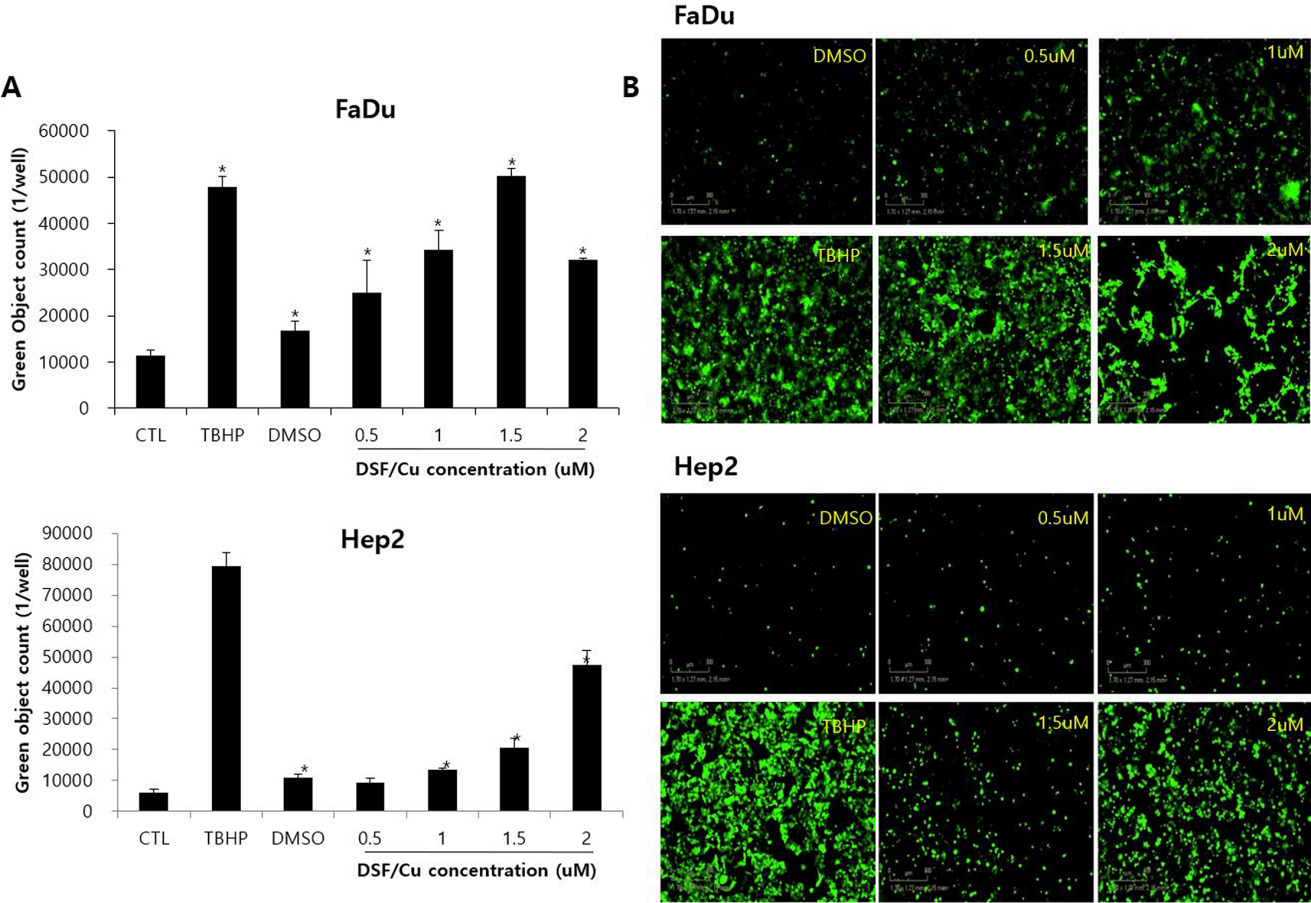
HNSCC cell lines were treated with DSF/Cu at various concentrations (0.5–2 μM) and ROS production was analyzed by Incucyte analysis after 24 hours. DSF/Cu induced ROS production in a dose-dependent manner in the HNSCC cell line. Statistically significant ROS formation induced by DSF was noted to start a concentration of 0.5 μM in FaDu cells and at a concentration of 1 μM in Hep2 cells. DMSO (negative control) and TBHP (positive control). *p<0.05.

HNSCC cell lines were treated with DSF/Cu at various concentrations (0.5–2 μM) and ROS production was analyzed by Incucyte assay 24 hours after starting DSF/Cu treatment. (Fig 2) DSF/Cu increased ROS production in a dose-dependent manner. In the FaDu cell line, DSF induced a statistically significant increase in ROS formation starting at a concentration of 0.5 μM. (p<0.05). In the Hep2 cell line, DSF induced a statistically significant increase in ROS formation at a concentration of 1 μM. (p<0.05).

### DSF induces cytotoxicity through autophagic cell death

**Fig 3:**
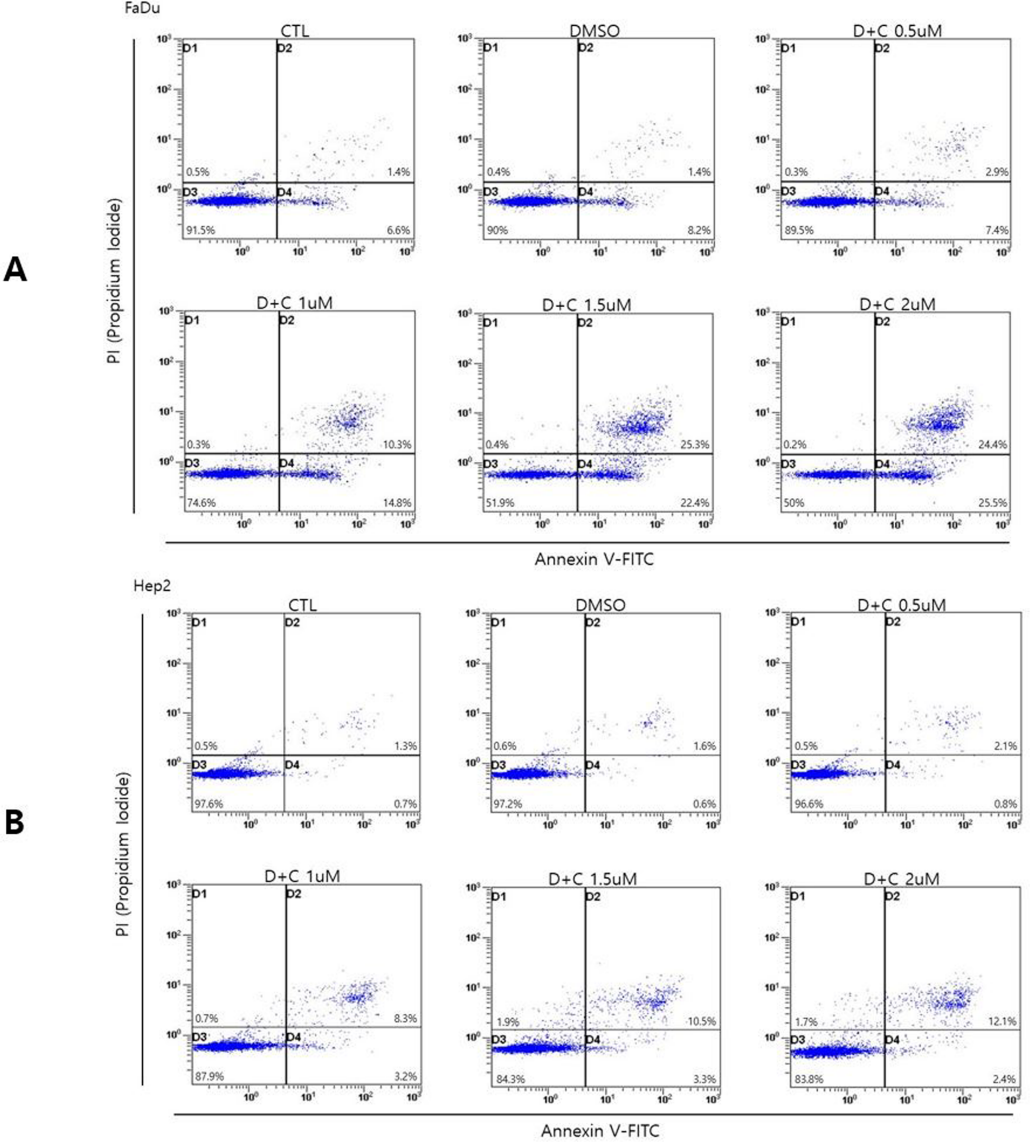
Apoptotic cell death induced by DSF/Cu treatment in head and neck cancer cells. Flow cytometry was used for quantification of DSF/Cu induced apoptosis: annexin V-fluorescein isothiocynate (FITC) and propidium iodide (PI) staining were used for analysis of the percentage of apoptotic cells treated with DSF/Cu. The percentages of apoptotic cells in DSF/Cu treated groups did not exceed 49% in FaDu cells (A) and 14% in Hep2 cells (B), even when treated with 2 μM DSF/Cu.

**Fig 4:**
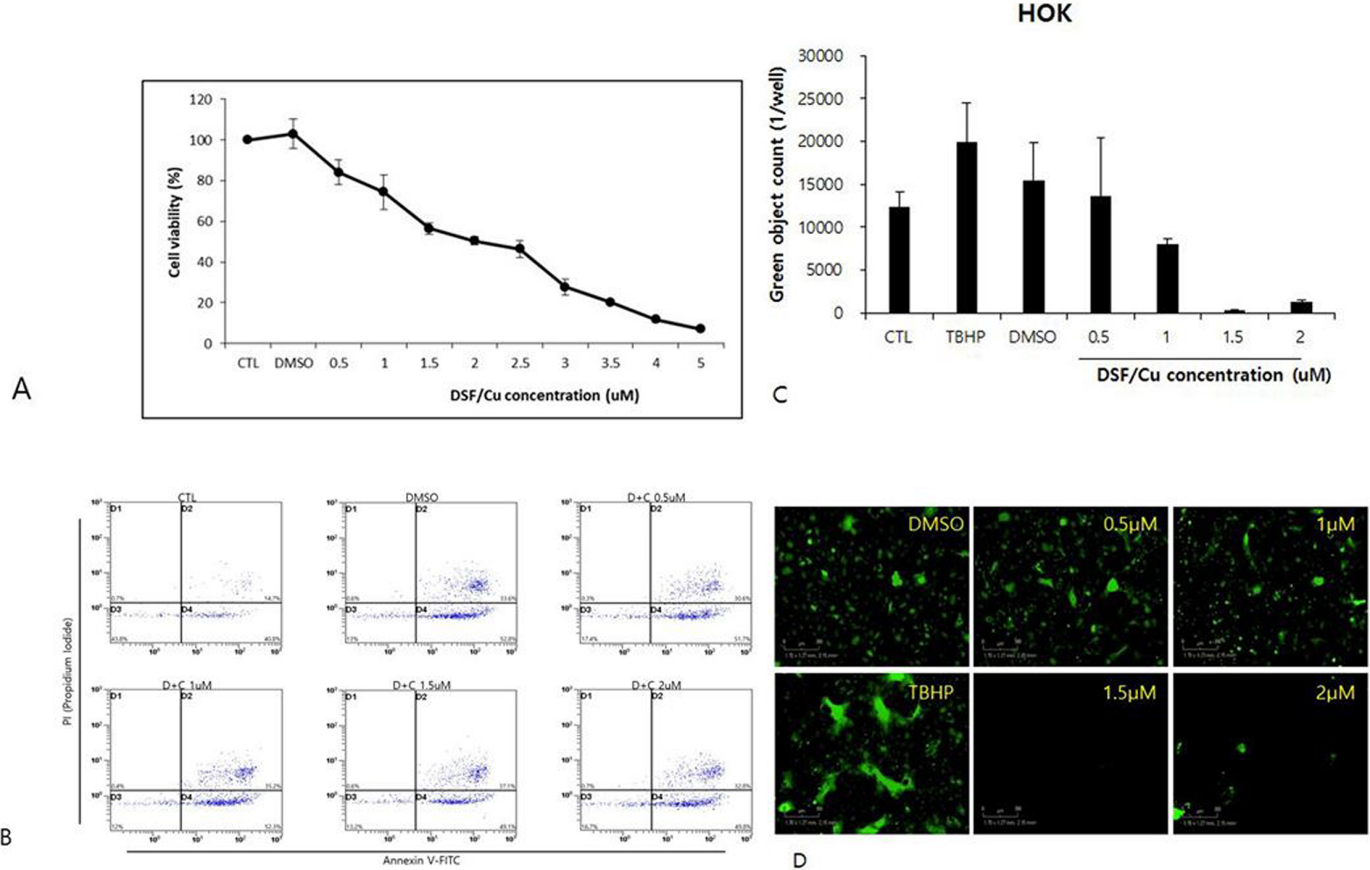
Oral normal keratinocyte was treated with DSF at the indicated concentrations (0.5–5 μM) and cell viability was measured by CCK-8 assay. A marked decrease in viability mediated by DSF was noted at 0.5 μg/mL in oral normal keratinocyte. Data are expressed as the mean ± standard deviation of three independent experiments. *p<0.05.

To confirm whether DSF/Cu induces apoptosis in HNSCC cell lines, we next performed Annexin V/PI fluorescence-activated cell sorting in HNSCC cell lines treated with DSF/Cu. Flow cytometry was used for quantification of DSF/Cu induced apoptosis: annexin V-fluorescein isothiocynate (FITC) and propidium iodide (PI) staining were used for analysis of the percentage of apoptotic cells treated with DSF/Cu. The percentages of apoptotic cells in DSF/Cu treated groups did not exceed 49% in FaDu cells (A) and 14% in Hep2 cells (B), even when treated with 2 μM DSF/Cu. (Fig 3) Additionally, we performed in vitro experiments with normal keratinocyte. In CCK8 assay, a marked decrease in viability mediated by DSF/Cu treatment was also noted at 0.5 μg/mL in normal keratinocyte. However, the percentages of apoptotic cells in DSF/Cu treated groups did exceed 80% in normal keratinocyte, even when treated with 0.5 μM DSF/Cu on annexin V/PI fluorescence-activated cell sorting. To measure ROS production in normal keratinocyte, Incucyte analysis was performed. However, unlike HNSCC cell lines (FaDu and Hep2), a significant increase of ROS production was not detected after DSF/Cu treatment compared to control. These results suggested that anti-cancer effect of disulfiram via autophagic cell death was unique to head and neck cancer cell lines, not for normal keratinocyte. (Fig 4)

**Fig 5:**
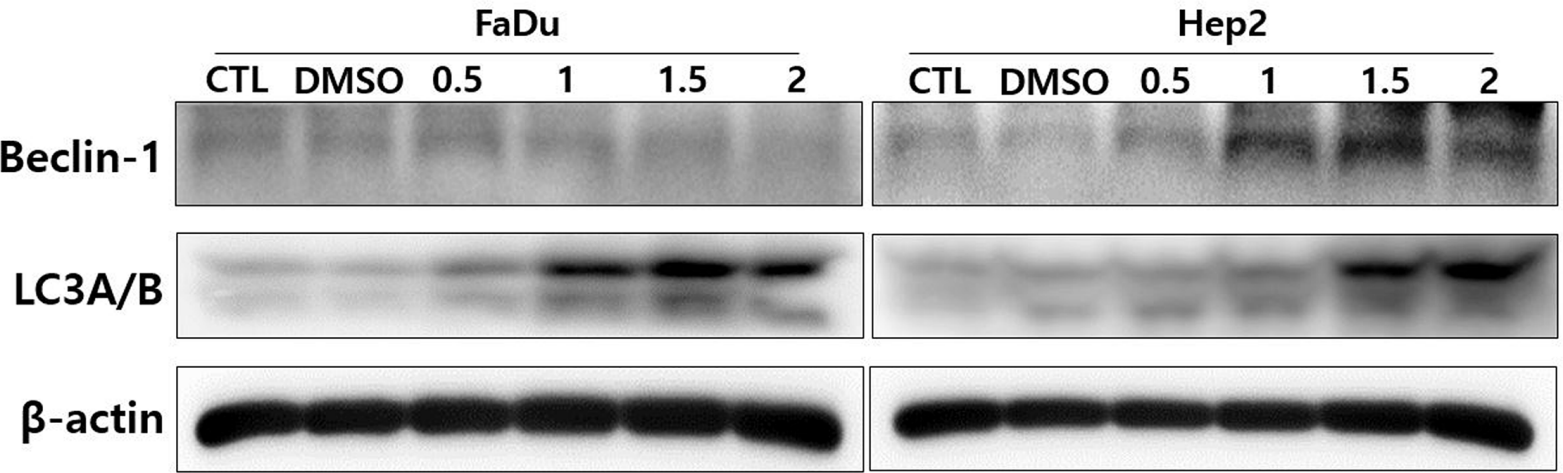
Western blot analysis showed that the expression of Beclin-1 and LC3B I/II, which are necessary for autophagosome formation, were increased by DSF/Cu after treatment of DSF/Cu on HNSCC cell lines. Beclin-1 and LC3B I/II were expressed in a dose-dependent manner.

**Fig 6:**
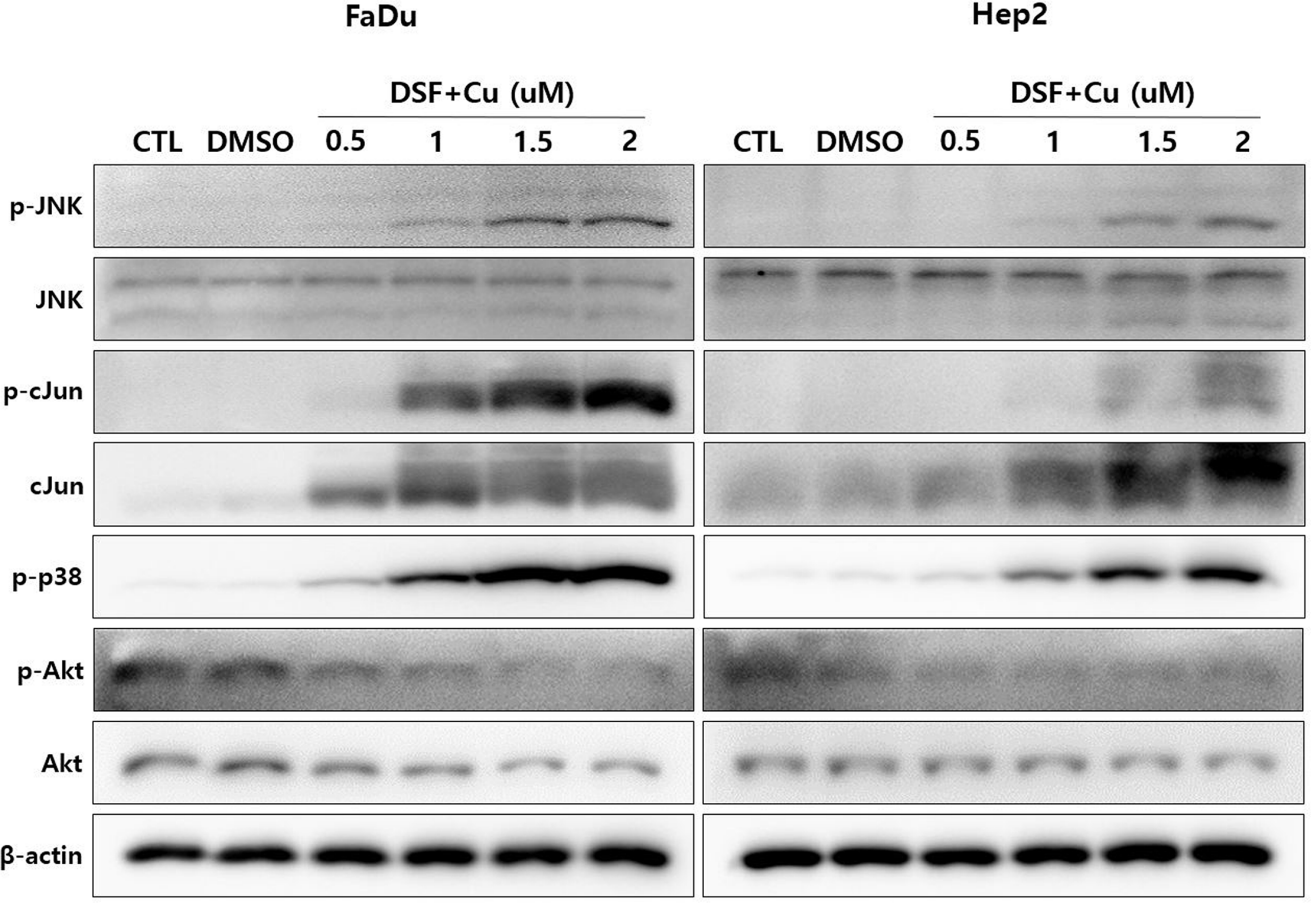
Western blot analysis investigating the effects of DSF/Cu on Akt signaling, which is known to be involved in autophagy. After DSF treatment, p-JNK and p-p38 were increased and phosphorylation of p-Akt was decreased. These results suggest that down-regulation of Akt signaling is involved in DSF/Cu-mediated autophagic cell death.

The above results suggested that cell death induced by DSF does not occur via apoptosis or necrosis, but by other mechanisms. Thus, we next investigated whether DSF/Cu-induced cell death was mediated through autophagy. Western blot analysis showed that the expression of Beclin-1 and LC3B I/II, which are necessary for autophagosome formation, were increased by DSF/Cu. (Fig 5) In addition, Western blot analysis showed that Akt signaling, which is known to be involved in autophagy, was inhibited by DSF/Cu. Specifically, levels of p-JNK and p-p38 were increased after DSF/Cu treatment while phosphorylation of p-Akt was decreased. These results suggested that down-regulation of the Akt signaling pathway may be involved in the induced of autophagic-mediated cell death by DSF/Cu. (Fig 6)

### DSF/Cu inhibits tumor progression in HNSCC xenograft model

**Fig 7:**
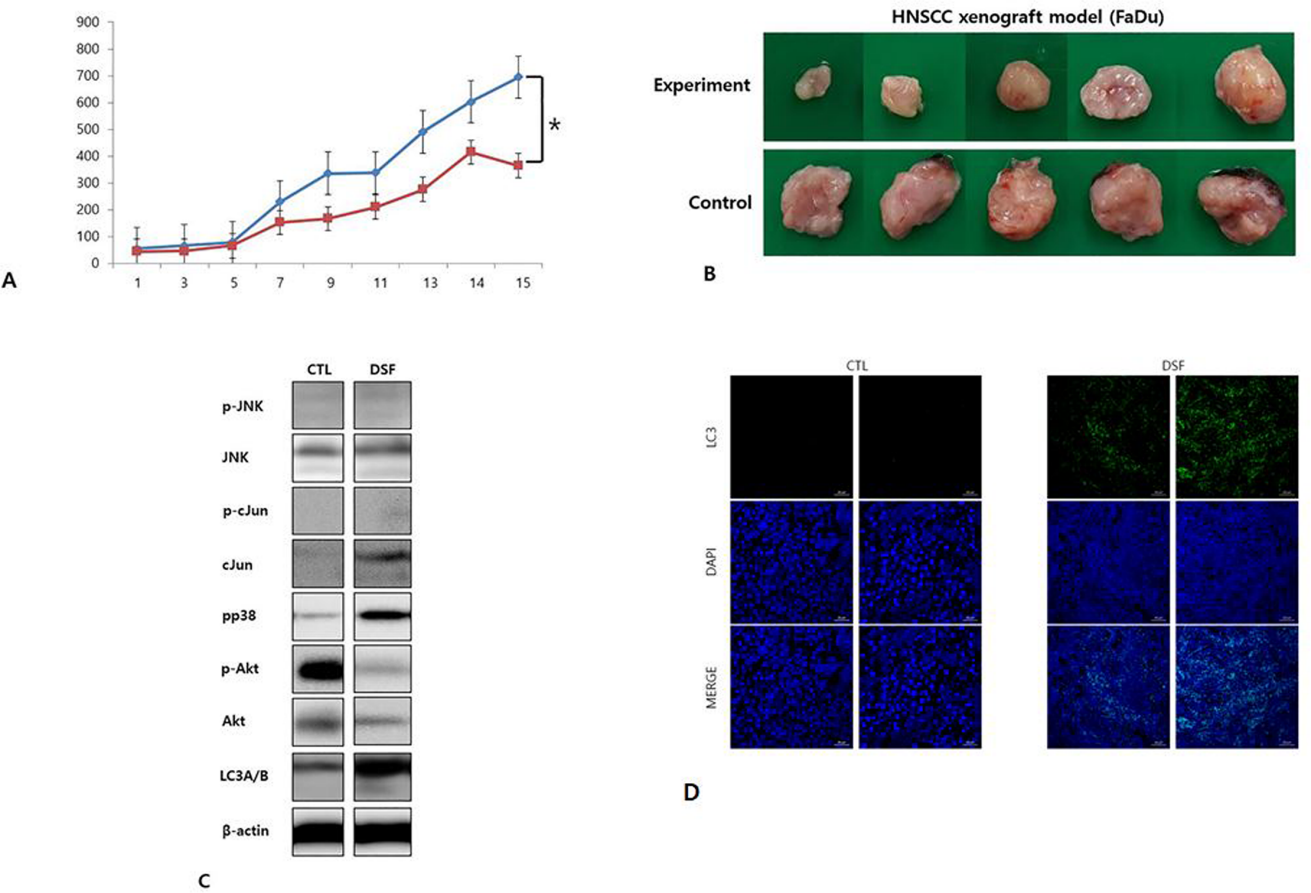
A and B. Xenograft experiment. Mice bearing FaDu tumors were treated with DSF/Cu (50 mg/kg/day) 5 days per week for 2 weeks and tumor growth was measured in the control and experimental groups. Tumor growth in the experimental group was significantly less than that of the control group. *p<0.05. C. Tumor tissues was tested by Western blot analysis. Akt signaling was suppressed in DSF treated group and LC3B I/II were significantly increased after DSF treated group. (CTL: control, DSF: DSF treated group) D. Tumor tissues were removed and stained with DAPI and LC3 antibody. Compared to control group (CTL), nuclear degradation was observed only in the experimental group (DSF) and the tumor tissues were strongly stained with LC3 only in the experimental group (DSF).

We next analyzed the effect of DSF/Cu on tumor growth in an HNSCC xenograft model. A total of 2x10^6^ FaDu cells were injected into the right flank of BALB/c nude female mice. After allowing tumors to form for 2 weeks, DSF/Cu was injected 5 days per week for 2 weeks, during which time tumor volumes were measured daily. Tumor growth was significantly inhibited in the experimental group injected with DSF/Cu compared to the control group. (Fig 7A and 7B) After euthanasia of these mice, tumor tissues were removed. Harvested tumor tissues were tested by Western blot analysis (Fig 7C) and stained with DAPI and LC3 antibody. On western blot analysis, phopho-Akt was suppressed and LC3B I/II were significantly increased in DSF treated group. Compared to control group (not DSF treated), nuclear degradation was observed only in the experimental group (DSF treated) and the tumor tissues were strongly stained with LC3 only in the experimental group (DSF treated). (Fig 7D)

## Discussion

This is the first study to confirm the anti-cancer effects and mechanism of DSF in HNSCC using in vitro and in vivo studies. Previous studies have shown that a single dose of 250 mg of DSF leads to a maximum serum concentration of 1.3 μM, and can reach 1.4 μM by repeated administration. [14] Therefore, we utilized DSF concentrations between 0.5 and 2.0 μM in order to maintain clinical relevance. In addition, since the physiological blood concentration of Cu in healthy subjects is known to be 10 μg / dl (1.6 μM/L), we adjusted the concentration of Cu used in our experiments to 0–2 μM as well. [15] The anti-cancer effects of DSF in HNSCC were also found to be dependent on Cu, which has been demonstrated in various malignancies. [16,17] DSF inhibited the effects of HNSCC in a Cu dependent manner, and the combination of DSF/Cu markedly inhibited HNSCC cells beginning at a concentration of 1 μM. ROS production was remarkable starting a DSF/Cu concentration of 0.5 μM, suggesting that the inhibitory effect of DSF/Cu on HNSCC is mediated through ROS formation. In the HNSCC cell lines, phospho-JNK, phospho-cJun and phospho-p38 were increased and phospho-Akt was decreased after DSF/Cu treatment. Together, these results suggested that the inhibitory effects on HNSCC after DSF/Cu administration involve both ROS formation, phosphorylation of JNK, and down-regulation of Akt signaling. Through these molecular mechanisms, DSF ultimately induce the inhibitory effects on HNSCC cell lines mainly through autophagic cell death, not apoptotic cell death. Lastly, we investigated the clinical relevance of our in vitro results using a xenograft animal model with FaDu cells. The results of our in vivo experiment showed that tumor growth was remarkably decreased by DSF (50 mg/kg) injection in an HNSCC xenograft model.

Alcohol intake increases the risk of developing HNSCC, and is also known to increase the risk of death related to HNSCC. [18] According to a multi-center study performed in Italy, sustained alcohol intake is a prognostic factor associated with decreased survival in patients with laryngeal cancer. [19] Therefore, proper counseling on alcohol consumption before and after treatment in patients with HNSCC is an important factor for determining patient prognosis. DSF inhibits the activity of acetaldehyde dehydrogenase in the liver, thereby inhibiting alcohol breakdown and resulting in the accumulation of acetaldehyde in the body. Such accumulation of acetaldehyde induces an unpleasant effect, prompting patients to avoid further alcohol consumption. Treating alcohol dependence using DSF along with appropriate counseling in HNSCC patients may contribute to an improvement in survival. Thus, when coupled with the anti-cancer effect of DSF on HNSCC revealed in our study, DSF may have important therapeutic potential in HNSCC patients with a dismal prognosis.

The tumor-suppressive effects of DSF are known to be due to inhibition of proteasome and suppression of cancer stem cells. Skrott et al. recently reported that the tumor suppressive effects of DSF appear to target NPL4, which is essential for the turnover of proteins involved in multiple regulatory and stress-response pathways in cancer cells. [20] In addition, Wang et al. reported that the suppression of ALDH2 after DSF/Cu administration reverses microtubule inhibitor resistance both in vitro and in vivo. [21] Furthermore, DSF inhibits ALDH1-positive cell subpopulations, which in turn can overcome resistance to cisplatin. [22] These unique anti-cancer effects of DSF may represent an alternative way to overcome resistance to chemotherapy and radiotherapy commonly observed in the treatment process of HNSCC patients. Further studies will be useful for identifying the precise molecular targets of DSF responsible for anti-cancer effects of HNSCC and investigating its efficacy in therapeutic-resistant HNSCC.
